# Effectiveness of One Videoconference-Based Exposure and Response Prevention Session at Home in Adjunction to Inpatient Treatment in Persons With Obsessive-Compulsive Disorder: Nonrandomized Study

**DOI:** 10.2196/52790

**Published:** 2024-03-13

**Authors:** Ulrich Voderholzer, Adrian Meule, Stefan Koch, Simone Pfeuffer, Anna-Lena Netter, Dirk Lehr, Eva Maria Zisler

**Affiliations:** 1 Schoen Clinic Roseneck Prien am Chiemsee Germany; 2 Department of Psychiatry and Psychotherapy University Hospital, LMU Munich Munich Germany; 3 Medical Center, Department of Psychiatry and Psychotherapy, Faculty of Medicine University of Freiburg Freiburg Germany; 4 Institute of Medical Psychology, Faculty of Medicine LMU Munich Munich Germany; 5 MindDoc Health GmbH Munich Germany; 6 Leuphana Universität Lüneburg Lüneburg Germany

**Keywords:** obsessive-compulsive disorder, videoconference-based treatment, therapy, exposure, response prevention, OCD, prevention, inpatient, video, videoconference, therapist, therapists, mood, positive mood, environment, clinical setting

## Abstract

**Background:**

Therapist-guided exposure and response prevention (ERP) for the treatment of obsessive-compulsive disorder (OCD) is frequently conducted within clinical settings but rarely at places where patients are usually confronted with OCD symptom-provoking situations in daily life (eg, at home).

**Objective:**

This study aimed to investigate patients’ views on 1 ERP session at home via videoconference and its impact on treatment outcome.

**Methods:**

A total of 64 inpatients with OCD received 1 session of therapist-guided videoconference-based ERP at home in adjunction to a multimodal inpatient treatment between 2015 and 2020.

**Results:**

Compared with 64 age- and sex-matched controls who received a multimodal inpatient treatment without 1 session of videoconference-based ERP at home, patients who received 1 session of videoconference-based ERP in adjunction to a multimodal inpatient treatment showed stronger reductions in OCD symptom severity from admission to discharge. Before the videoconference-based ERP session, patients reported high rationale credibility and treatment expectancy. After the videoconference-based ERP session, patients reported medium-to-high positive mood as well as depth and smoothness of the session, and they perceived the working alliance as high.

**Conclusions:**

Results highlight the importance of administering therapist-guided ERP sessions in patients’ natural environment to enhance treatment response in OCD. Videoconference-based ERP as add-on to treatment as usual is, therefore, a promising approach to facilitate the application of ERP in patients’ natural environment and foster the generalization of ERP conducted in clinical settings.

## Introduction

### Background

Obsessive-compulsive disorder (OCD) is a mental disorder characterized by intrusive and disturbing thoughts as well as repetitive patterns of behavior [1,2]. These are often multifaceted, that is, they include different obsessions and compulsions related to unwanted intrusive thoughts, fears of diseases, and contamination, among others [2,3]. OCD is a common disorder with a lifetime prevalence ranging from 1% to 3% and often has debilitating consequences on the daily functioning, well-being, and quality of life of affected persons as well as family members [4,5]. It usually emerges in late adolescence or early adulthood and has a chronic course if effective treatment is lacking [6,7]. Yet, OCD is often underrecognized and missed in primary care settings [8]. Thus, the duration of untreated illness in adults often exceeds 10 years, which creates a large treatment gap [9].

### Exposure and Response Prevention in the Treatment of OCD

Cognitive-behavioral therapy (CBT) with exposure and response prevention (ERP) is the first-line, evidence-based psychotherapeutic treatment for OCD and is recommended as the psychotherapeutic method of choice [10-12]. ERP is a crucial element in CBT for OCD and requires patients to “engage in repeated, prolonged exposure to obsessions while refraining from compulsions” ([13], p. 85) [14]. Recent evidence stemming from inhibitory learning theory suggests that patients learn new associations during ERP (eg, “dirt—no fatal disease”), which in turn inhibit existing maladaptive associations (eg, “dirt—fatal disease” [15]). This acquisition of associations is enabled by expectancy violation that is a mismatch between patients’ expectancy and outcome [15]. Although ERP is particularly useful in reducing OCD symptomatology, many patients find it difficult to endure upcoming unpleasant cognitions, feelings, and bodily sensations [16].

Besides the challenging nature of ERP itself, patients with OCD receiving CBT with ERP have to face a variety of difficulties [16]. First, patients are required to understand the underlying principles and measures of a treatment that is referred to as rationale credibility [17]. Second, patients need to expect that the treatment they are undergoing is effective [17-19]. Finally, it is beneficial if a positive working alliance is established between the patient and psychotherapist as it appears to predict treatment outcome [20]. Despite patients having to face various challenges when undergoing ERP, this psychotherapeutic intervention is highly effective for many people with OCD [21].

### Home-Based ERP

The most commonly applied form of ERP is therapist-guided ERP in clinical settings (eg, at inpatient wards and in offices of psychotherapists), although the intervention can possibly be provided in several ways and facilities [22]. However, as persons with OCD often face the occurrence of obsessions and compulsions at home and feared situations or triggers cannot be replicated in a hospital or office, it can be hypothesized that home-based ERP may be beneficial in the treatment of OCD [23]. Although the theoretical framework of administering ERP at patients’ homes may sound reasonable, evidence on this treatment variant is mixed. Although some studies found that ERP at home was slightly advantageous in terms of symptom reduction [24,25], others reported that home-based ERP was no more effective than standard office-based ERP [23].

There are a variety of reasons why home-based ERP is not administered on a regular basis by the majority of psychotherapists. Specifically, many clinicians lack time or familiarity with this intervention [22,26,27]. Additionally, specialized hospitals are not always located close to the patient’s home, making therapist-guided ERP in the patient’s living environment difficult to conduct. Even with outpatient therapy close to the patient’s home, there is the challenge of therapists having to travel to the patient’s place, which is difficult to implement due to limited time resources of therapists. Therefore, patients often receive outpatient treatment that only includes a limited number of therapist-assisted exposure sessions, if any [22,28].

To achieve a better care situation for patients with OCD, there are 2 cost-effective ways of implementing therapist-guided home-based ERP. The first option is telephone-supported ERP, which was shown to be effective in 2 studies [29,30]. With advancing technologies, the second option is videoconference-based ERP, which can also be considered an adequate tool that comes with significant reductions in obsessive-compulsive symptoms, especially in persons with moderate OCD symptoms [27,31-33]. Videoconference-based psychotherapy has several advantages over in-person psychotherapy. First, by using videoconference-based psychotherapy, treatment with ERP can easily be delivered to patients who are homebound or living in rural areas [31,34]. Second, the administration of home-based in vivo exposures allows the generalization of treatment effects to other contexts [31,34]. Third, the therapist is in charge of accompanying and supporting the patient during ERP [35]. Fourth, therapist-assisted ERP has been shown to be more effective than non–therapist-assisted ERP [36], and using videoconference at home might allow for even more therapist-assisted ERP.

### This Study

As research on videoconference-based ERP is still limited, we examined treatment effects in patients who received inpatient treatment with an additional videoconference-based ERP at home compared with an age- and sex-matched group of patients who received inpatient treatment without an additional videoconference-based ERP at home. Second, we assessed patients’ views on the current intervention (ie, treatment expectancy and rationale credibility) before undergoing the videoconference-based ERP session. Third, we examined patients’ evaluations of the videoconference-based ERP session (ie, depth, smoothness, positivity, and arousal) and working alliance with the therapists after having received videoconference-based ERP. We expected stronger reductions in OCD symptom severity in patients who received inpatient treatment with an additional videoconference-based ERP session at home compared with inpatients who received multimodal inpatient treatment without an additional videoconference-based ERP session at home from admission to discharge. Furthermore, we expected high ratings on rationale credibility and treatment expectancy before as well as high ratings on satisfaction with the therapeutic sessions and quality of the therapeutic relationship from the patients’ perspective after the videoconference-based ERP session at home.

## Methods

### Sample Characteristics

This study was a nonrandomized, 2-group design study, in which a subset of patients who voluntarily participated in the study (videoconference exposure group) were compared with another subset of patients who did not participate in the study (control group). Although this design has disadvantages compared with a randomized controlled trial (RCT; see the *Discussion* section), it can be conducted more conveniently (eg, is less expensive and requires less resources) and may even have higher external validity as randomization may influence participation and outcomes when patients have a treatment preference [37]. Inpatients with OCD treated at the Schoen Clinic Roseneck (Prien am Chiemsee, Germany) between 2015 and 2020 were investigated. In Germany, inpatient treatment is indicated if at least 1 of the following factors applies: absence of or nonresponse to guideline-based disorder-specific outpatient treatment, danger to life, severe neglect, compulsive and avoidant behavior that is either severe or habitual resulting in an inability to maintain a normal daily routine and adherence to outpatient treatment, severe suffering and impairment of psychosocial functioning, psychological or somatic comorbidities aggravating outpatient treatment, and a particularly disease-promoting environment [10,38]. The treatment provided at the Schoen Clinic Roseneck adheres to the German S3 guidelines for the treatment of OCD [10]. Thus, the therapeutic concept is multimodal and consists of symptom-specific, individual CBT and ERP sessions, and other treatment elements, depending on indication (eg, psychopharmacological medication; see Table 1).

**Table 1:** Sample characteristics (N=128).

Characteristic			Videoconference exposure group (n=64)		Control group (n=64)		Test statistics													
							Chi-square (*df*)			*U*			*V*			*P* value			*r*_*rb*_ (*d*)	
**Subtype of obsessive-compulsive disorder (ICD-10^a^ code), n (%)**								1.62 (N/A^b^)			N/A			0.11			.44			N/A
	Obsessions-only subtype (F42.0)	0 (0)		1 (2)																
	Compulsions-only subtype (F42.1)	8 (13)		11 (17)																
	Mixed subtype (F42.2)	56 (88)		52 (81)																
Sex (female), n (%)			42 (66)		46 (72)		0.58 (N/A)			N/A			0.07			.45			N/A	
Age (years), mean (SD)			26.95 (12.26)		29.28 (13.78)		N/A			1888.00			N/A			.45			–0.08 (–0.18)	
Length of stay (days), mean (SD)			93.33 (30.54)		85.88 (38.40)		N/A			2302.50			N/A			.23			0.12 (0.22)	
**Any comorbid mental disorder, n (%)**			44 (69)		43 (67)		0.04 (N/A)			N/A			0.02			.85			N/A	
	Affective disorders	32 (50)		34 (53)		0.13 (N/A)			N/A			0.03			.72			N/A		
	Anxiety disorders	11 (17)		16 (25)		1.17 (N/A)			N/A			0.10			.28			N/A		
	Eating disorders	6 (9)		4 (6)		0.43 (N/A)			N/A			0.06			.51			N/A		
Antidepressant medication^c^, n (%)			28 (49)		23 (51)		0.04 (N/A)			N/A			0.02			.84			N/A	
Total score of Obsessive-Compulsive Inventory—Revised at admission, mean (SD)			31.56 (12.62)		31.32 (14.89)		N/A			2095.00			N/A			.83			0.02 (0.02)	
Total score of Yale-Brown Obsessive-Compulsive Scale at admission, mean (SD)			23.63 (5.33)		22.57 (6.51)		N/A			2202.50			N/A			.46			0.08 (0.18)	

^a^ICD-10: International Classification of Diseases, Tenth Revision.

^b^N/A: not applicable.

^c^Information missing for 7 patients in the videoconference exposure group and 19 patients in the control group.

A total of 88 inpatients participated in this study, that is, received 1 videoconference-based ERP session at home in addition to inpatient treatment. As inpatient treatment at the Schoen Clinic Roseneck consists of 3 phases (psychoeducation and motivation, ERP, and transfer to the patients’ homes), participating patients were in the third phase of inpatient treatment. Psychotherapists at the hospital who had undergone technical training on videoconference-based ERP were authorized to offer the intervention to their patients. Patients were free to choose whether or not to receive the additional videoconference-based ERP session at home. On average, persons who received videoconference-based ERP at home had moderate OCD symptom severity according to the self-report version of the Yale-Brown Obsessive-Compulsive Scale (Y-BOCS; mean sum score 23.63, SD 5.33; Table 1; see recommendations by Cervin et al [39]).

Inpatients with OCD who were treated at the hospital within the same time period but who did not receive a videoconference-based ERP session at home were selected as the control group. Yet, these patients also received therapist-guided ERP in the hospital. Similar to the persons having received the videoconference-based ERP session, persons in the control group had, on average, moderate symptom severity according to the Y-BOCS (mean sum score 22.57, SD 6.51; Table 1; see recommendations by Cervin et al [39]). At the Schoen Clinic Roseneck, data from diagnostic assessments (eg, age, sex, diagnoses, medication, length of stay, and questionnaire scores) are automatically transferred to a database from which they can be exported without any identifying information by authorized employees. Thus, accessing individual patient charts is not necessary.

**Figure 1:**
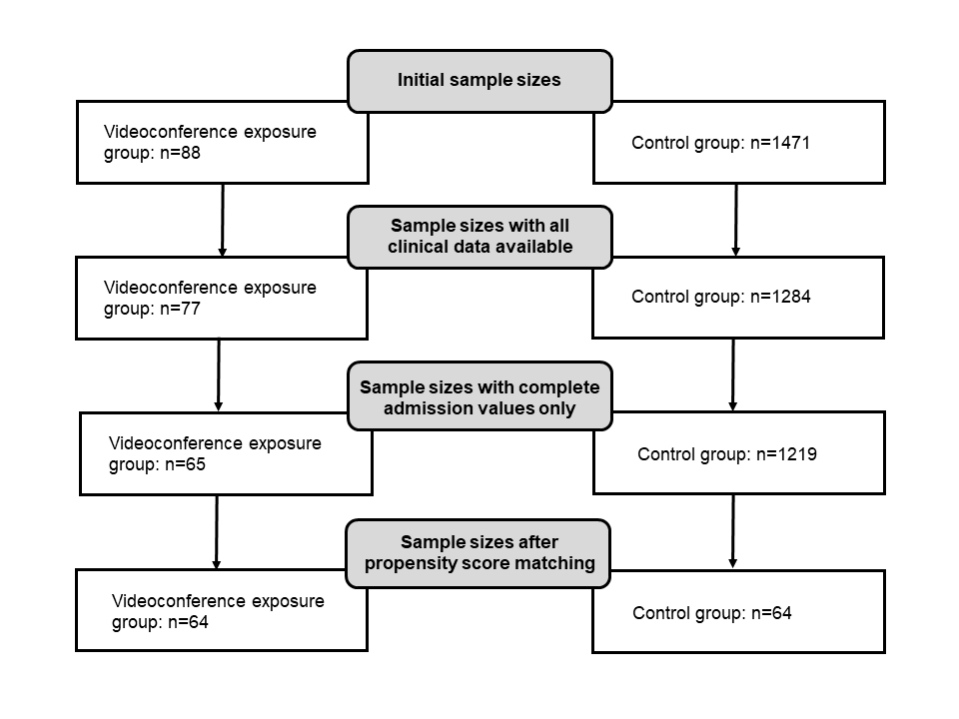
Participant flowchart.

Between 2015 and 2020, a total of 1471 patients with OCD were treated in the hospital who did not receive videoconference-based ERP at home, that is, did not take part in the study. Because of missing data, 1219 patients were available for matching with 65 of the 88 patients in the videoconference exposure group (Figure 1). Groups were matched based on propensity score matching without replacement using the FUZZY extension for SPSS (version 27.0; IBM Corp) [40]. Data were matched in regard to the variables age, sex, any comorbidity, length of stay, Obsessive-Compulsive Inventory–Revised (OCI-R) scores at admission, and Y-BOCS scores at admission. Using a match tolerance with which all 65 persons in the videoconference exposure group were retained did not result in well-matched groups (ie, groups still differed in age and length of stay). Thus, a match tolerance of 0.019 was chosen, which led to the exclusion of 1 person from the videoconference exposure group, resulting in a final sample size of 128 (ie, 64 persons per group; Table 1).

### Measures

#### OCI-R Questionnaire

The OCI-R [41,42] was used to examine obsessive-compulsive symptoms. The OCI-R is an 18-item self-report questionnaire with 6 subscales: washing, checking, ordering, obsessing, hoarding, and neutralizing. Responses are recorded on a 5-point scale ranging from 0 (not at all) to 4 (extremely) and refer to the extent of distress during the past month due to OCD symptoms. In a previous study, internal reliability coefficients for the 6 subscales ranged between α=.76 and .95. In this study, the internal reliability coefficient for the total scale was ω=0.82 at admission and ω=0.86 at discharge.

#### Y-BOCS Questionnaire

The self-report version [43] of the Y-BOCS [44,45] was used to examine OCD severity. The Y-BOCS is a 10-item self-report questionnaire comprising 2 subscales: obsessions and compulsions. Responses are recorded on a 5-point scale ranging from 0 (no symptoms) to 4 (extreme symptoms)*.* Internal reliability coefficients ranged between α=.78 and .88 in 2 validation studies [46,47] and between ω=0.83 and 0.91 in this study. Convergent validity has been supported by high correlations with other measures for obsessive-compulsive symptomatology, and divergent validity has been supported by moderate correlations with measures for related but distinct constructs such as worry [48-50].

#### Credibility Expectancy Questionnaire

The Credibility Expectancy Questionnaire (CEQ) [17] was used to assess the rationale credibility and treatment expectancy of the patient. The CEQ is a 6-item self-report questionnaire with 2 subscales: rationale credibility and treatment expectancy. Responses are recorded on a 9-point scale ranging from 1 (not at all) to 9 (very much)*.* Internal reliability coefficients for the subscales ranged between ω=0.71 and 0.88.

#### Session Evaluation Questionnaire

The Session Evaluation Questionnaire (SEQ) [51] was used to examine the patients’ satisfaction with the therapeutic sessions. The SEQ is a 21-item self-report questionnaire with 4 subscales: depth, smoothness, positivity, and arousal. Responses are recorded on a 7-point scale ranging from 1 (unpleasant) to 7 (pleasant). Internal reliability coefficients for the subscales ranged between ω=0.61 and 0.87. A closer inspection revealed that 2 items (1=slow, 7=fast; 1=moved, 7=composed) contributed to a low internal reliability of the arousal subscale. After removing those items, the remaining items of the arousal subscale had an internal reliability of ω=0.76. Thus, internal reliability coefficients for the subscales then ranged between ω=0.76 and 0.87.

#### Working Alliance Inventory—Short Revised

The Working Alliance Inventory—Short Revised (WAI-SR) [52] was used to examine the quality of the therapeutic relationship from the patient’s perspective. The WAI-SR is a 12-item self-report questionnaire with 3 subscales: task, goal, and bond. Responses are recorded on a 7-point scale ranging from 1 (never) to 7 (always)*.* Internal reliability coefficients for the subscales and the total scale ranged between ω=0.84 and 0.88.

### Procedure

The videoconference app “VidyoMobile” by Vidyo, Inc was used to enable visual and auditory communication between the patient and therapist [25]. Patients were taught by a research staff member on how to use the smartphone, the tripod, and the videoconference app. Moreover, therapists prepared ERP sessions with their patients in close detail in a preceding session in the hospital. Before the ERP session, patients completed the CEQ. Patients received 1 videoconference-based ERP session each at home either on Friday afternoon or Monday morning. Each session had a duration of 2 hours on average. All videoconference-based ERP sessions were conducted by therapists specialized in CBT and ERP, and only the patient and the therapist were attending the session. The primary goal of the videoconference-based ERP session was to practice difficult situations associated with obsessions and compulsions in the patient’s home. The therapist’s role was to encourage the patient to face upcoming unpleasant feelings, emotions, and bodily sensations and to accompany them emotionally [16]. The exact execution of actions during ERP (ie, turning off the stove without checking, washing hands only once, etc) was not controlled by the therapist so as to give the patient a sense of personal responsibility in their own home.

After the ERP session, patients completed the SEQ and WAI-SR. In addition, after the videoconference-based ERP session, patients were asked to continue practicing the exposure exercise on their own. These exercises were not accompanied by the therapist, but debriefing followed in subsequent therapy sessions. Questionnaires assessing symptom severity (ie, OCI-R and Y-BOCS) were completed by the patients at admission and discharge.

### Data Analyses

Group differences on categorical variables (OCD subtype, sex, comorbid mental disorders, and antidepressant medication) were tested with χ^2^ tests and on continuous variables (age, length of stay, and questionnaire scores at admission) with Mann-Whitney *U* tests. Due to missing data at discharge (OCI-R: n=28, Y-BOCS: n=26), we examined changes of OCI-R and Y-BOCS total scores from admission to discharge as a function of a group with robust linear mixed models, which include cases with missing data in the maximum likelihood estimation. For this, we used R [53] and RStudio [54] and, specifically, the R package *robustlmm* [55]. The 2 models (1 for OCI-R scores and 1 for Y-BOCS scores) included fixed effects of time (admission vs discharge), group (videoconference exposure group vs control group), and their interaction term as well as a random intercept (ie, person-level random variability in scores at admission). As the package *robustlmm* does not produce parameter-specific *P* values, we used the workaround by Geniole et al [56]. Specifically, nonrobust models were fitted with the *lme4* package [57], *P* values were obtained with the package *lmertest* [58], and Satterthwaite-approximated degrees of freedom generated by the *lme4* models were combined with the output of the *robustlmm* model [56,59].

### Ethical Considerations

The study was approved by the ethics committee of the Psychological Department of the Philipps University of Marburg, Germany. According to the guidelines by the institutional review board of the LMU Munich, retrospective analyses on already available anonymized data are exempt from requiring ethics approval. All participants in the videoconference exposure group signed informed consent before taking part in the study.

## Results

**Figure 2:**
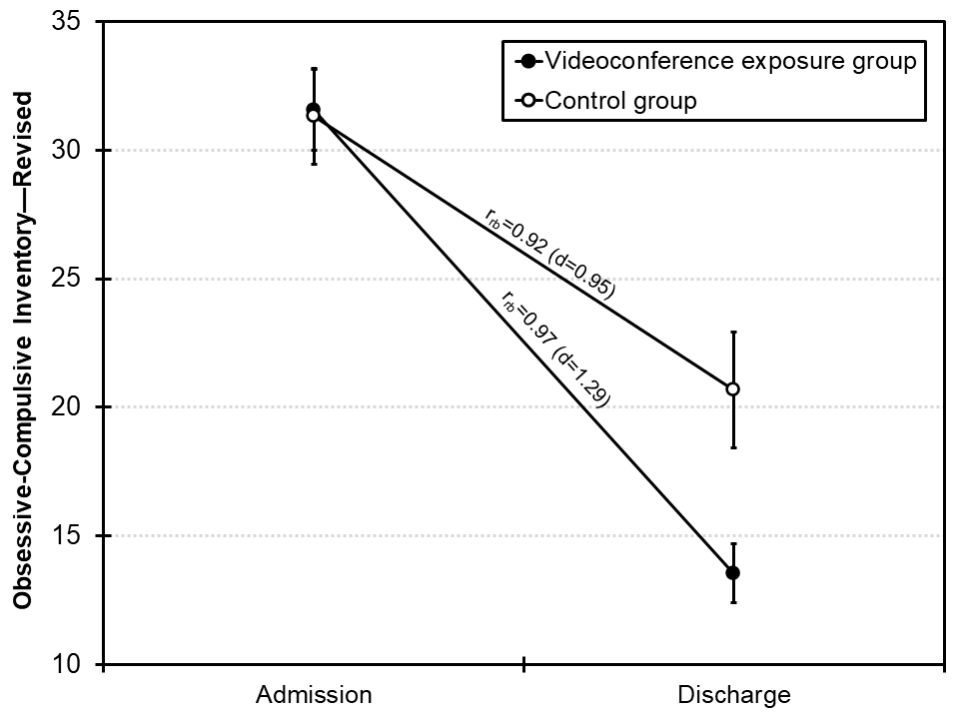
Mean sum scores of the Obsessive-Compulsive Inventory—Revised at admission and discharge as a function of group. The error bars indicate the SE of the mean. Effect sizes (rank biserial correlation coefficients r_rb_ and Cohen d) refer to the changes within each group from admission to discharge.

**Figure 3:**
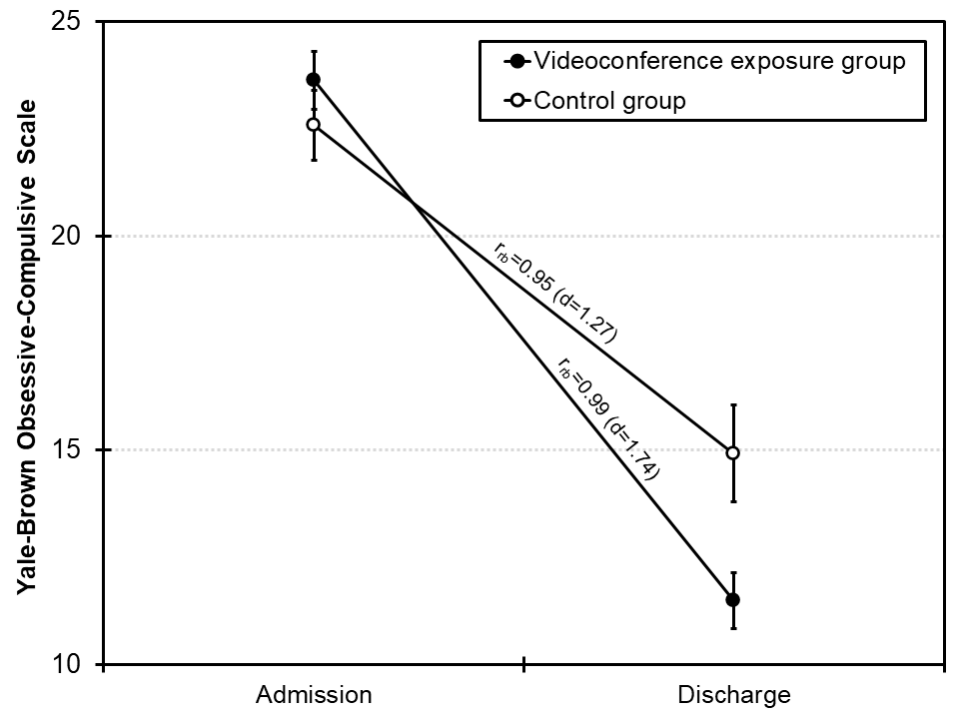
Mean sum scores of the Yale-Brown Obsessive-Compulsive Scale at admission and discharge as a function of group. The error bars indicate the SE of the mean. Effect sizes (rank biserial correlation coefficients r_rb_ and Cohen d) refer to the changes within each group from admission to discharge.

As can be seen in Table 1, both groups did not significantly differ in age, sex, having any comorbid mental disorder, OCD subtype, antidepressant medication, OCI-R total scores at admission, and Y-BOCS total scores at admission. Robust linear mixed models revealed statistically significant interactions for group time for OCI-R (*b=*6.27; *P=*.01) and Y-BOCS (*b*=4.58; *P*<.001) scores, indicating that OCD symptom changes from admission to discharge differed as a function of group. As can be seen in Figures 2 and 3, the videoconference exposure group had larger OCD symptom reductions from admission to discharge than the control group. Descriptive statistics for obsessive-compulsive symptoms (total scores for OCI-R and Y-BOCS) at admission and discharge in the videoconference exposure and control groups are displayed in Table 2. On a scale ranging from 1 to 9, patients had mean (SD) values of 8.03 (0.74) on the subscale *rationale credibility* and 7.24 (1.13) on the subscale *treatment expectancy* on the CEQ. On a scale ranging from 1 to 7, patients had mean (SD) values of 5.87 (0.97) on the subscale *depth*, 3.60 (1.29) on the subscale *smoothness*, 4.61 (1.43) on the subscale *positivity*, and 4.11 (1.30) on the (reduced) subscale *arousal* on the SEQ. On a scale ranging from 1 to 7, patients had mean (SD) values of 6.25 (0.65) on the subscale *therapeutic tasks*, 6.52 (0.60) on the subscale *therapeutic goals*, 6.34 (0.75) on the subscale *therapeutic bond*, and 6.37 (0.57) on the total scale of the WAI-SR.

**Table 2:** Descriptive statistics for obsessive-compulsive symptoms at admission and discharge in the videoconference exposure and control groups.

Time point and statistic		Videoconference exposure group				Control group		
		n (%)	Mean (SD)	Range	n (%)		Mean (SD)	Range
**Admission**								
	Obsessive-Compulsive Inventory—Revised	64 (50)	31.56 (12.62)	6-56	64 (50)		31.32 (14.89)	3-59
	Yale-Brown Obsessive-Compulsive Scale	64 (50)	23.63 (5.33)	9-36	64 (50)		22.57 (6.51)	5-35
**Discharge**								
	Obsessive-Compulsive Inventory—Revised	57 (44.5)	13.54 (8.59)	1-35	43 (33.6)		20.67 (14.81)	3-56
	Yale-Brown Obsessive-Compulsive Scale	56 (43.8)	11.48 (4.88)	1-25	46 (35.9)		14.92 (7.67)	1-31

## Discussion

### Summary of Results

This study showed that the group that had an additional therapist-guided, videoconference-based ERP session at home showed greater improvements during inpatient treatment for OCD, that is, displayed larger decreases in OCD symptomatology compared with treatment as usual. Obsessive-compulsive symptoms from admission to discharge decreased for patients who received a videoconference-based ERP session at home as well as for patients who received treatment as usual without a videoconference-based ERP session with medium to large effect sizes. Yet, obsessive-compulsive symptoms decreased even stronger for patients who have received inpatient treatment and a videoconference-based ERP session as an add-on. Furthermore, patients had high treatment expectancy and perceived the rationale as credible before receiving videoconference-based ERP. After undergoing videoconference-based ERP, patients perceived depth (ie, potency and value), smoothness of the session (ie, comfort and relaxation), and mood after the session (ie, positivity and arousal) as medium to high. Patients who received videoconference-based ERP rated working alliance (ie, agreement on therapeutic tasks and goals as well as therapeutic bond) with their therapist as high.

### Possible Mechanisms of Videoconference-Based ERP-Enhanced Symptom Reductions

Our results revealed that patients who received videoconference-based ERP at home in adjunction to a multimodal inpatient treatment had higher symptom reductions from admission to discharge with higher effect sizes than the control group. This might be significantly attributable to patients being able to generalize and extend their progresses achieved in the hospital to their own home; that is, with the help of the personal support of their therapist, they are more successful in giving up avoidance behavior at home as well [60]. Yet, it must be considered that there was no randomization in this study, which is why factors other than the additional videoconference-based ERP session might have also contributed to the reduction in OCD symptomatology from admission to discharge.

Alternative explanations for higher OCD symptom reductions in the videoconference exposure group might be that mostly patients who were highly motivated decided to participate in the additional videoconference-based ERP session or that the psychotherapists who treated patients receiving videoconference-based ERP were more motivated compared with other psychotherapists who treated the other patients with traditional ERP in the hospital only. Additionally, it might be possible that psychotherapists themselves expected that the additional ERP session at home would be beneficial for the patients and, thus, were highly engaged in the therapeutic sessions in the hospital as well, which particularly helped patients in reducing their OCD symptoms.

Despite methodological restrictions in nonrandomized study designs such as this study, there are also several disadvantages in RCTs that must be taken into account. First, participants are no passive recipients of interventions and do have treatment preferences. Patients with specific treatment preferences might, thus, refuse to take part in RCTs to avoid being randomized to the nonpreferred treatment, which reduces external validity [37]. Second, patients included in RCTs are strongly preselected, which was not the case in this study. Thus, the characteristics of patients included in this study correspond more to the real care situation. Third, internal validity of RCTs could be reduced as randomization to the nonpreferred treatment might influence patient adherence to the treatment protocol [37]. Accordingly, as this study was a nonrandomized study, patients were able to express and act on their treatment preferences as they could choose to receive the additional videoconference-based ERP session at home. This might have substantially increased patient adherence, which could, in turn, have been a factor contributing to reductions in OCD symptomatology. Furthermore, the 2 groups in this study were matched based on propensity score matching, which aims to account for absent randomization as it imitates some of the characteristics of an RCT [61]. Propensity score matching helps to strengthen causal arguments in observational studies by reducing selection bias [62].

Besides significant reductions in OCD symptomatology from admission to discharge in patients in the videoconference exposure group, the current results indicate that patients mainly had positive views on the videoconference-based ERP session, which became apparent in positive subjective ratings of the sessions. The positive effects of the videoconference-based ERP session on OCD symptomatology might be due to several change factors (ie, treatment expectancy and working alliance) that appear to be targeted in the videoconference setting to a sufficient degree. Several studies have provided evidence that treatment expectancy and understanding of the underlying treatment rationale are powerful predictors of psychotherapy outcome in general [18]. Additionally, as patients rated working alliance in the videoconference setting as high, this might also substantially contribute to the effects shown in this study. Previous studies have already shown that the videoconference setting enables the patient and psychotherapist to establish a strong and stable working alliance that is comparable to that in traditional face-to-face treatment [63,64]. Several studies even highlight that a positive working alliance is predictive of substantial decreases in symptomatology [31]. Although this study cannot show causal associations between working alliance and symptom reductions, a positive working alliance might substantially be linked to improvements of the patients’ condition in the face-to-face and videoconference setting.

### Limitations

As in every study, interpretation of the current results is limited to the persons and methods investigated. First, the examination of obsessive-compulsive symptoms was based on self-report, and—although the instruments used (OCI-R and Y-BOCS) are characterized by high validity and reliability—future studies may include therapist-rated measurements (eg, Y-BOCS interview version, Clinical Global Impression-Improvement Scale, and Global Assessment of Functioning [65,66]) as the inclusion of multiple views on the patients’ OCD symptomatology allows for an even more comprehensive evaluation. Second, due to limited material and human resources in the hospital, only a subset of patients treated at the hospital received an additional videoconference-based ERP session at home. Therefore, future studies might make the treatment available to a larger sample and replicate the effect. Third, future studies might examine the effects of multiple videoconference-based ERP sessions as the current add-on intervention included only 1 ERP session. Fourth, there was no randomization in this study. Hence, there might also be a number of factors other than the additional videoconference-based ERP session at home that might have contributed to significant OCD symptom reductions (eg, motivation to engage in ERP might have differed between the 2 groups and different therapists administered ERP sessions). Thus, conducting RCTs is recommended for future studies.

### Conclusions

Altogether, this study showed that the group that received a 1-time home visit of videoconference-based ERP in adjunction to a multimodal inpatient treatment had greater improvements, that is, larger decreases in OCD symptomatology, during inpatient treatment of OCD compared with treatment as usual. In addition, patients’ ratings showed that the videoconference setting as well as working alliance with therapists was largely perceived as pleasant. Overall, it is recommended to provide patients with OCD with therapist-guided ERP at home. If it is not possible to accompany the intervention in person due to time constraints or other issues, videoconference-based therapy is a promising alternative to facilitate the application of ERP in patients’ natural environment and foster the generalization of treatment effects achieved in clinical settings.

## Abbreviations

CBT: cognitive-behavioral therapy
CEQ: Credibility Expectancy Questionnaire
ERP: exposure and response prevention
OCD: obsessive-compulsive disorder
OCI-R: Obsessive-Compulsive Inventory—Revised
RCT: randomized controlled trial
SEQ: Session Evaluation Questionnaire
WAI-SR: Working Alliance Inventory—Short Revised
Y-BOCS: Yale-Brown Obsessive-Compulsive Scale

## Data Availability

The data sets generated during and/or analyzed during this study are available in the Open Science Framework repository (https://osf.io/pybhw/).
